# Response of Metastatic Chordoma to the Immune Checkpoint Inhibitor Pembrolizumab: A Case Report

**DOI:** 10.3389/fonc.2020.565945

**Published:** 2020-12-17

**Authors:** Xiaoli Wu, Xiangwu Lin, Ying Chen, Wencui Kong, Jinhe Xu, Zongyang Yu

**Affiliations:** ^1^ Fu Zong Clinical Medicine, Fujian Medical University, Fuzhou, Fujian, China; ^2^ Department of Medical Oncology, The 900th Hospital of the Joint Logistic Support Force, PLA, Fuzhou, China; ^3^ Department of Respiratory and Critical Care Medicine, Dongfang Hospital of Xiamen University, Fuzhou General Hospital of Fujian Medical University, The 900th Hospital of the Joint Logistic Support Force, PLA, Fuzhou, China

**Keywords:** chordoma, immunotherapy, PD-1, pembrolizumab, *PBRM1* gene mutation

## Abstract

Chordoma is a rare primary bone tumor that exhibits insensitivity to radiotherapy and chemotherapy and has a poor prognosis. Currently, resection is the primary treatment for affected patients, but the subsequent rate of recurrence is high, and both overall survival (OS) and progression-free survival (PFS) are consequentially relatively short. This case report describes a patient who was diagnosed with metastatic chordoma that was found to possess the A1209fs mutation of the *PBRM1* gene, which may be associated with beneficial responses to immunotherapies. The patient received pembrolizumab, an immune checkpoint inhibitor (ICI) that targets the PD-1 receptor of lymphocytes, as second-line therapy, which he tolerated well (the most frequent adverse events were abnormal liver function and hyperglycemia, both of which were only grades 1–2), and achieved a PFS duration of 9.3 months. We hope these results will promote further research that will clarify the mechanisms underlying this beneficial response and that will further explore the use of immunotherapies in this population.

## Background

Chordoma is a rare primary bone tumor that is derived from embryonic notochordal remnants. These tumors are most frequently observed at the base of the skull and the sacrococcygeal area, which are located at opposite ends of the spine. The incidence of this rare disease is approximately 1/1,000,000 per year (1). The main symptom of chordoma is local pain, which is usually caused by the invasion and compression of adjacent structures and depends on the location of the lesion. These tumors are insensitive to radiotherapy and chemotherapy; therefore, surgery remains the most effective treatment among patients, with complete resection being the most significant prognostic tool. Adjuvant radiotherapy, such as proton therapy and carbon ion therapy, have been showed to be quite effective for chordomas. Data from the US showed that between 1973 and 2009, the average survival of chordoma patients who underwent surgery and/or radiation was 6.29 years (2), while in China 2017, the median progression-free survival (PFS) was 6.0 years and the median overall survival (OS) was 9.6 years (3). Though the OS of patients with chordoma is relatively long, this tumor has strong local invasiveness. Thirty percent of chordomas develop distant metastases, and more than half of chordoma patients suffer postoperative recurrence; moreover, once the disease relapses, the outcome of patients is very poor (4).

No chemotherapy is currently approved for the treatment of chordoma, and only a few targeted drugs, including Imatinib and Dasatinib, are recommended, based on small-sample studies (5, 6). A retrospective case series analysis of 46 patients with advanced chordoma treated with Imatinib showed that the best response was stable disease, which was achieved in 34 (74%) patients, and that the PFS and OS were 9 and 34 months, respectively (7). Forty-two patients (87.5%) reported toxicity of any grade, and the most frequently reported adverse events were edema, increased creatinine levels, and gastrointestinal side effects. In addition, in other advanced chordomas, the PFS rate at 6 months was 33% when patients were treated with nitro-camptothecin (8). None of these represent attractive options.

In other cancers found to be nonresponsive to chemotherapies, such as melanoma, alternative approaches, including immunotherapies, have been found to be effective (9). However, the efficacy of PD-1-/L1-based immunotherapy for chordoma patients remains poorly understood. As one of the main targets of currently available immunotherapies, PD-L1 is highly expressed in chordoma cell lines, suggesting that PD 1/L1-based immunotherapy may have potential in the treatment of chordoma (10). To the best of our knowledge, only two patients with advanced chordoma treated with PD-1-based immunotherapy have been described worldwide in the literature, and both of these patients achieved impressive clinical and radiological responses (11).

Here, we report a case of a chordoma patient who was negative for PD-L1 expression in the original tumor and found to possess the A1209fs mutation of the *PBRM1* gene. *PBRM1* is located on chromosome 3 and encodes a protein that is a subunit of the PBAF form of the SWI/SNF chromatin remodeling complex. The loss-of-function mutations in the *PBRM1* gene often relate to immune checkpoint inhibitor (ICI) responses. The A1209fs mutation of the *PBRM1* gene may be associated with beneficial responses to immunotherapies, so pembrolizumab was selected as a treatment for this patient.

## Case Presentation

**Figure 1:**
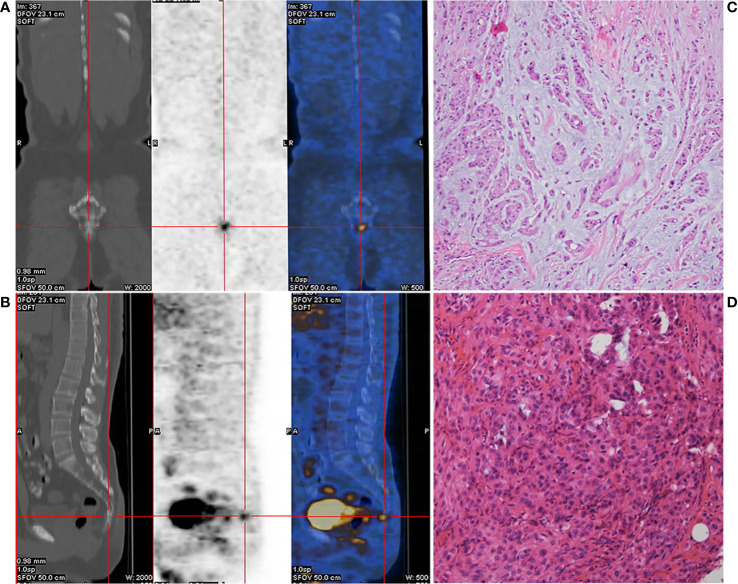
Preoperative positron emission tomography/computed tomography (PET-CT) of the caudal vertebra and histological features of the chordoma. Coronal section **(A)** and median sagittal section **(B)** of the PET-CT in chordoma. Pathologic findings from the initial surgical resection specimen (**C, D**, HE ×400).

A 52-year-old male patient with lumbosacral pain was first admitted to the First Affiliated Hospital of Fujian Medical University in November 2017. Positron emission tomography/computed tomography (PET-CT) imaging revealed slight distension of and a concentration of radiation in his coccyx 1 (S1) vertebra (**Figures 1A, B**). The patient received complete resection of the primary tumor. A surgical specimen obtained from the S1 vertebra was pathologically diagnosed as chordoma (**Figures 1C, D**). Immunohistochemistry (IHC) showed that the tumor was positive for epithelial membrane antigen, vimentin, and cytokeratin 8/18, 5% positive for Ki-67, and negative for glial fibrillary acidic protein, S-100 protein, and E-cadherin (data not shown). The patient did not receive adjuvant therapy. In August 2018, follow-up imaging showed multiple nodules considered to be metastases in both lungs. Computed tomography (CT)-guided needle biopsy was subsequently performed and showed that the metastases were positive for epithelial membrane antigen, vimentin, and cytokeratin 8/18 and 20% positive for Ki-67 (data not shown). According to the patient’s medical history, imaging examination results, and IHC findings, he was diagnosed with metastatic chordoma.

Next-generation sequencing (NGS) was performed on formalin-fixed, paraffin-embedded (FFPE) surgical tissue sections (ACT Genomics Co., Ltd.; mean depth, 1574+; uniformity 93%), which showed that the primary tumor had the A1209fs mutation of the *PBRM1* gene and a low tumor mutational burden (TMB) (6.9 mut/Mb. The FFPE samples were negative for PD-L1 expression.

**Figure 2:**
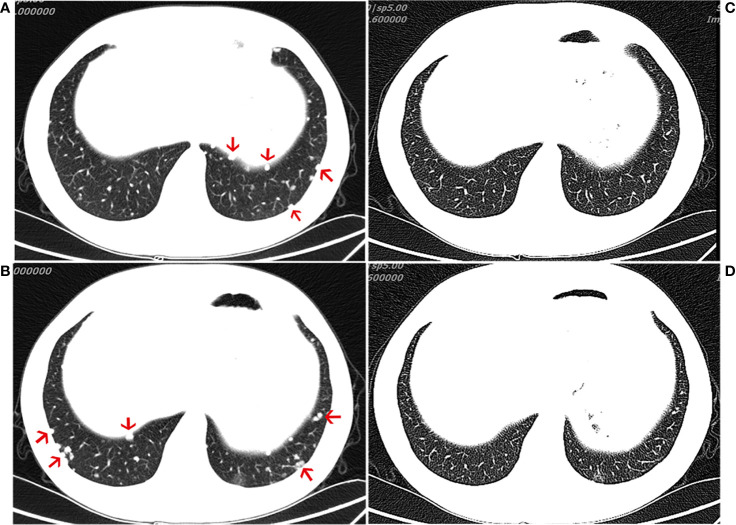
Computed tomography (CT) of the chest. CT scans showing the chest **(A, B)** before immunotherapy and 4 month(s) after the initiation of immunotherapy treatment **(C, D)**, demonstrating a partial response.

The patient was treated with everolimus (self-medication) for 2 months, and repeat CT imaging showed no reduction in the metastases. The patient was then referred to our hospital, where he started treatment with the ICI, pembrolizumab 200 mg every 3 weeks, in November 2018. During the course of regular treatment with pembrolizumab, the most frequent adverse events were abnormal liver function and hyperglycemia. Both of these adverse events were only grades 1–2 and observed late in the clinical course. After 4 months of treatment, the patient exhibited an obvious reduction in the number of metastatic nodules and achieved a partial response (PR) (evaluated by the modified Response Evaluation Criteria in Solid Tumors [iRECIST] for immunotherapy trials, which defines PR as a reduction of more than 30% in tumor load) (**Figure 2**). After 11 cycles of pembrolizumab, imaging showed multiple bone metastases. The clinical response lasted for 9.3 months until progression occurred in August 2019, leading to the discontinuation of pembrolizumab. The patient continues to undergo treatment and follow-up.

## Discussion

The immune microenvironment of chordoma remains poorly understood. Of the 78 chordoma samples from 56 patients, 94.9% were positive for PD-L1 expression. Tumors with positive PD-L1 expression were significantly associated with advanced stages of chordoma and the median PD-L1 expression score for the metastatic group was significantly higher than that for the primary group, suggesting that PD 1/L1-based immunotherapy may have potential in the treatment of chordoma, especially in advanced patients (10, 12).

Here, we present the 3rd case of a patient with advanced chordoma (in this case with negative PD-L1 expression and a low TMB in the original tumor specimen) treated with anti-PD1 antibodies and show that similar to the previous two cases, our patient also showed a good antitumor response (**Figure 2**). These three cases seem to suggest that immunotherapies could represent an alternative to Imatinib in advanced chordoma. There are very few cases of chordoma in general, and even those have shown slight efficacy; therefore, such therapies may be helpful. In our case, following the initiation of immunotherapy, imaging showed that a favorable response was achieved at 4 months (**Figure 2**). Although it is not clear whether metastatic tumors express PD-L1, whether chordoma patients with low PD-1/L1 expression would benefit from this particular immunotherapy remains to be further studied. A variety of clinical trials are underway to explore the effects of immunotherapies in chordoma (NCT02989636 and NCT03623854), and we look forward to the new findings produced by these trials.

In reviewing the literature, we discovered that our patient had unique characteristics, including a low TMB and the A1209fs mutation of the *PBRM1* gene, that have not been previously reported in this condition. A high TMB indicates the presence of tumors that are likely to harbor neoantigens, making them targetable by activated immune cells (13). The low TMB observed in our case seemed to indicate limited tumor immunogenicity. However, the biological features of the original primary tumor may differ from those of the metastasis. Similar to PD-L1 expression, TMB in the metastatic tumor may also differ from that in the original tumor. Primary tumor PD-1/L1 expression and TMB are probably not definitive predictors of immunotherapy. The patient’s tumor also possessed the A1209fs mutation in the *PBRM1* gene. It has been reported that the loss of PBRM1 may benefit the efficacy of and enhance responses to immunotherapy due to the enrichment of immunostimulatory genes (including genes involved in the hypoxia response and JAK-STAT signaling) (14). Although the A1209fs mutation of the *PBRM1* gene has not been characterized in the literature, it results in a change in the amino acid sequence that likely causes the premature truncation of the functional PBRM1 protein. The results obtained in our case demonstrate that this truncation may be associated with a clinical benefit. Thus, we recommend further exploration of the potential therapeutic mechanisms of this gene mutation in chordoma.

## Conclusion

We administrated pembrolizumab (200 mg) every 3 weeks to a metastatic chordoma patient that was found to harbor A1209fs mutation of the *PBRM1* gene. The treatment achieved disease control, with a PFS duration of 9.3 months, suggesting that ICIs may be an attractive option for chordomas and calling for more research on immunotherapy for these tumors. The detection of PD-L1 in metastatic chordoma is particularly important for treatment decisions, regardless of the level of PD-L1 expression in the primary tumor. Mutations of the *PBRM1* gene may represent a new target of immunotherapies in chordoma and hence the need for further exploration.

## Ethics Statement

Ethical review and approval was not required for the study on human participants in accordance with the local legislation and institutional requirements. The patients/participants provided their written informed consent to participate in this study. Written informed consent was obtained from the individual(s) for the publication of any potentially identifiable images or data included in this article.

## Author Contributions

XW (first author) and XL contributed equally to the writing of the manuscript and designed the figures. YC and WK performed the clinical management of the patient. ZY (corresponding author) reviewed and edited the manuscript. JX collected the data. All authors contributed to the article and approved the submitted version.

## Funding

This work was supported by grants from the Clinical Key Specialty Construction Project of Fujian Province (Grant/Award Number: 2017YZ0001-2) and The 900th Hospital of the Joint Logistic Support Force of China: International Cooperative Research Program(Grant/Award Number: 2016G02).

## Conflict of Interest

The authors declare that the research was conducted in the absence of any commercial or financial relationships that could be construed as a potential conflict of interest.
